# Pre‐ and post‐skeletal muscle biopsy quantitative magnetic resonance imaging reveals correlations with histopathological findings

**DOI:** 10.1111/ene.16479

**Published:** 2024-09-16

**Authors:** Anne‐Katrin Güttsches, Johannes Forsting, Moritz Kneifel, Robert Rehmann, Alice De Lorenzo, Elena Enax‐Krumova, Martijn Froeling, Matthias Vorgerd, Lara Schlaffke

**Affiliations:** ^1^ Department of Neurology BG‐University Hospital Bergmannsheil, Ruhr‐University Bochum Bochum Germany; ^2^ Department of Neurology, Heimer Institute for Muscle Research BG‐University Hospital Bergmannsheil Bochum Germany; ^3^ Department of Radiology University Medical Centre Utrecht Utrecht Netherlands

**Keywords:** diffusion tensor imaging, histology, muscular diseases, quantitative magnetic resonance imaging, skeletal muscle

## Abstract

**Background and purpose:**

Quantitative muscle magnetic resonance imaging (MRI) is a promising non‐invasive method in the diagnostic workup as well as follow‐up of neuromuscular disorders. The aim of this study was to correlate quantitative MRI (qMRI) parameters to histopathological changes in skeletal muscle tissue and thus to verify the data from our pilot study.

**Methods:**

Twenty‐six patients (eight females, 46.4 ± 15.1 years) were examined within 72 h before and within 24 h after a skeletal muscle biopsy using quantitative muscle MRI. Post‐biopsy MRI was employed to pinpoint the exact localization of the biopsy. qMRI parameters including fat fraction, water T2 relaxation time and diffusion metrics including fractional anisotropy, mean diffusivity, axial diffusivity and radial diffusivity were extracted from the localization of the biopsy and correlated with histopathological findings. Additionally, three different segmentation masks were applied to the qMRI dataset, to evaluate whether the whole muscle represents the exact biopsy location.

**Results:**

Fat fraction and water T2 relaxation time in qMRI correlated significantly with the fat fraction in the muscle biopsy and histopathological inflammatory markers. Fractional anisotropy correlated with the quantity of type 2 fibres, whilst mean diffusivity correlated with p62. No differences were found using different segmentation masks in qMRI.

**Conclusions:**

In this follow‐up study, the results from our previous study were verified regarding the correlation of qMRI parameters with histopathological features in muscle biopsies, indicating that qMRI serves as a suitable non‐invasive method in the follow‐up of patients with neuromuscular disorders. If post‐biopsy MRI is not available, whole muscle volume can be used for histopathological correlations.

## INTRODUCTION

Skeletal muscle biopsy, accompanied with histopathological analysis, serves as an important tool for diagnosing neuromuscular disorders, particularly in distinguishing between inflammatory and hereditary myopathies [1]. Because skeletal muscle biopsy entails an invasive surgical procedure to obtain an adequate muscle tissue sample, it is not conducive to monitoring disease activity or assessing treatment outcomes. Therefore, in recent years quantitative muscle magnetic resonance imaging (MRI) has been developed which already presents a valuable non‐invasive tool in the evaluation of muscular injuries and neuromuscular disorders (NMDs) [2, 3, 4].

Chemical‐shift‐based water–fat separation techniques (further labelled as ‘Dixon‐based sequences’) are well established and widely used for calculating muscle fat fraction (FF) in muscle groups and single muscles, which is particularly valuable in assessing and monitoring NMDs [5, 6, 7]. An increase in the FF of muscle tissue, as measured by Dixon‐based sequences, has been observed to detect muscle pathology before clinical involvement of lower extremity muscles, making it a potential outcome parameter in clinical trials [8, 9]. Water T2 relaxation time (T2) is elevated in fluid retention and therefore has been hypothesized to reflect inflammation and myoedema [10, 11]. It has been shown that water T2 relaxation time has a significantly higher sensitivity in detecting early‐stage muscle abnormalities than the subjective grading of T2 turbo inversion recovery magnitude images [10]. Furthermore, muscle diffusion‐weighted imaging and analysis using diffusion tensor imaging can offer supplementary insights into both microstructures and macrostructures of muscle fibres. It can be used to reconstruct fibre tracts based on the diffusion properties of water molecules which are restricted by muscle fibre boundaries. Therefore, these reconstructed fibre tracts can depict the muscle fibre architecture [12, 13]. Changes in diffusion metrics are associated with varying mechanisms in different NMDs. In Becker's muscular dystrophy, diffusion‐weighted models of fibre diameter and size demonstrated a strong correlation with increased laminin fibre diameter in skeletal muscle biopsy [14]. In spinal muscular atrophy, a longitudinal study observed a decrease of fractional anisotropy (FA) in low‐fat‐replaced muscles, possibly indicating early muscle pathology, whilst other high‐fat muscles deteriorated [15]. In limb girdle muscular dystrophy, changes in diffusion parameter changes, including an increase of FA and decrease of radial diffusivity (RD) in non‐fat‐infiltrated muscles, were hypothesized as signs of early fibre degeneration, as supported by muscle biopsy findings [16].

As mentioned, quantitative MRI (qMRI) parameters capture distinct histopathological changes associated with different diseases. Using classic semiquantitative MRI sequences it was demonstrated that semiquantitative grading of fat infiltration correlated with histopathological abnormalities in needle and open muscle biopsy [17, 18]. Furthermore, Dixon‐based sequence‐derived FF has exhibited a good correlation with the histologically described degree of fat infiltration in different settings in animal models and humans [19, 20, 21]. Nevertheless, the relationships between water T2 relaxation time and diffusion metrics and histopathological findings remain elusive. Recent studies have provided the first indications that prolonged water T2 relaxation time may be linked to variations in myofibre size, inflammatory cell infiltration and the amount of connective tissue [19, 20]. Moreover, diffusion metrics have been shown to reflect tissue damage after exercise, as assessed by needle biopsy [22]. However, comprehensive evidence establishing correlations between histopathological findings and qMRI metrics is still sparse. Considering these facts, the present study aims to investigate correlations between qMRI metrics and histopathological findings within lower extremity muscles through a prospective cohort study.

In this follow‐up study, a naïve patient cohort was thus investigated, different from our pilot study. This was done also to evaluate whether the results of the pilot study can be reproduced in a larger cohort. The analysis of the quantitative outcome measures was identical, and in addition the exact biopsy localization could be identified by performing a post‐biopsy MRI, which allowed the qMRI data to be compared to the exact biopsy data. Thus, a major limitation of our previous study could be addressed, in which no post‐biopsy MRI was performed.

## METHODS

### Study population

**FIGURE 1:**
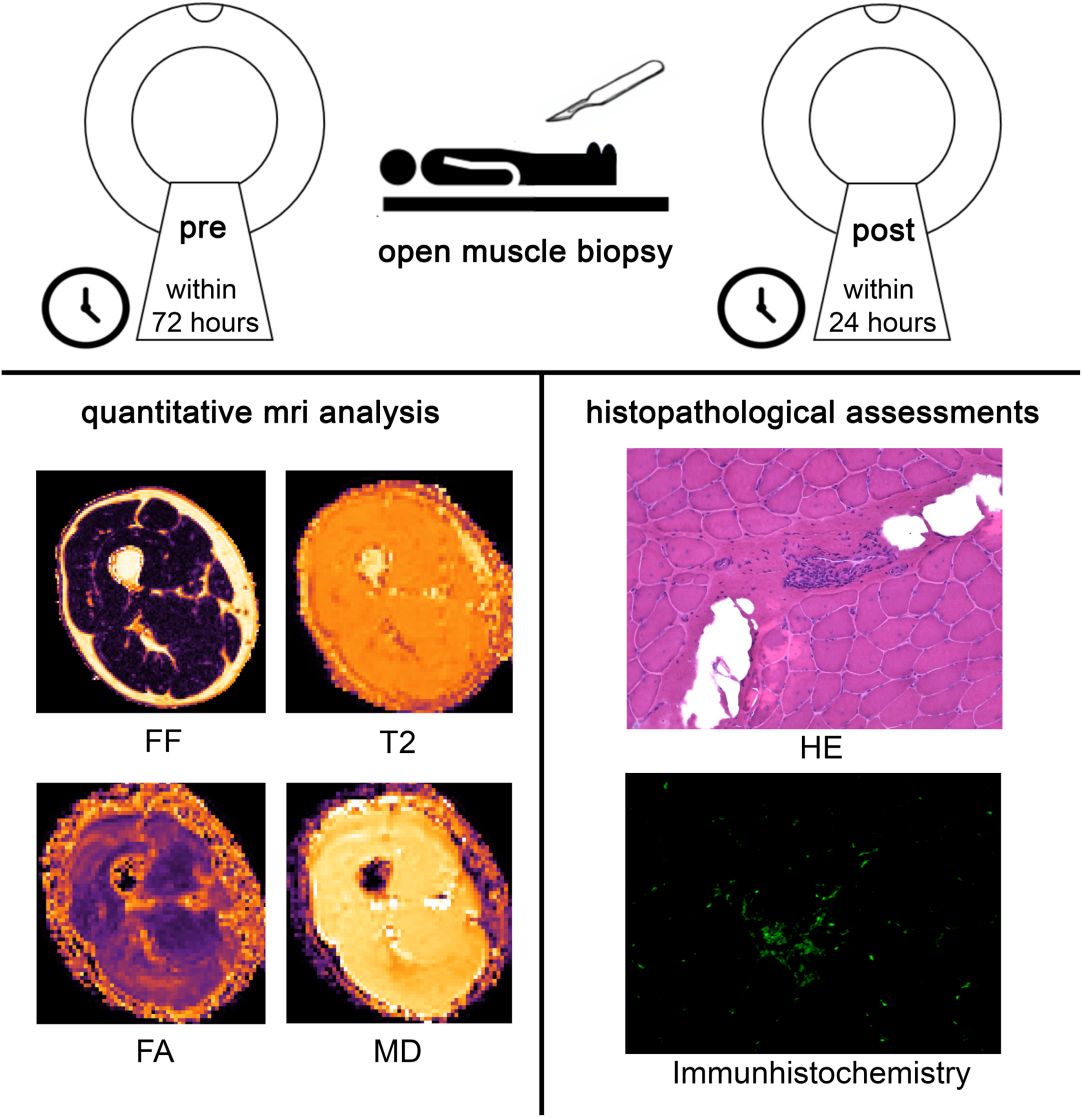
Study design of correlation analysis of quantitative MRI (qMRI) parameters and histopathological findings.

This prospective study was performed according to local ethics committee regulations and approved by the local Ethics Committee of the Ruhr University Bochum (reg. no. 20‐7080). Written consent was obtained from all participants. Twenty‐six patients (eight females, 46.4 ± 15.1 years, body mass index 27.2 ± 4.1) were examined within 1–3 days before and 6–24 h after a skeletal muscle biopsy using quantitative muscle MRI (see Figure 1). The overall patient recruiting and examination for this study was done between 2021 and 2023. The first patient was included in January of 2021 and the last patient was included in October of 2023. Patients were consecutively included in the study. The inclusion criterion was the conduction of a skeletal muscle biopsy during clinical workup; the exclusion criterion was the inability to be examined by MRI (i.e., due to magnetic implants or devices, claustrophobia or any other contraindication to performing an MRI). Table 1 gives an overview of patient clinical data.

**TABLE 1:** Clinical information of patients undergoing muscle biopsy.

No.	Sex	Age	BMI	Clinical complaint	Age at symptom onset	CK	Biopsy site	Diagnosis
1	M	59	28.3	Progressive myalgia, stress intolerance	34	82	Left GM	Unspecific changes
2	M	58	29.5	Proximal muscle weakness, myalgia	54	148	Left VL	Mitochondrial myopathy
3	F	73	29.8	Proximal muscle weakness	68	227	Right VL	Mitochondrial myopathy
4	M	38	32.4	Hyper‐CKemia, myalgia	33	403	Left GM	Neuromyopathy
5	M	32	31.2	Proximal muscle weakness, myalgia	24	261	Right VL	Unspecific changes
6	F	46	18.8	Myalgia, fatigue	36	311	Right VL	Polymyositis
7	F	55	24.6	Proximal muscle weakness	54	325	Left VL	Myofibrillar myopathy
8	M	39	28.7	Hyper‐CKemia, myalgia	39	883	Right VL	Necrotizing myopathy
9	M	76	24.6	Proximal muscle weakness	68	484	Left VL	Inclusion body myositis
10	F	20	23.9	Hyper‐CKemia, myalgia	18	1364	Left GM	Myositis
11	F	62	27.3	Muscle weakness, myalgia	55	48	Right GL	Inclusion body myositis
12	M	34	25.7	Myalgia	33	73	Left VL	Dermato‐ myositis
13	M	43	33.2	Myalgia	42	89	Right VL	Unspecific changes
14	F	55	23.2	Proximal muscle weakness	52	711	Left VL	Degenerative vacuolar myopathy
15	M	62	21.4	Acute proximal muscle weakness	61	7857	Left GM	Necrotizing myopathy
16	F	24	24.5	Hyper‐CKemia, myalgia	15	120	Right VL	Unspecific changes
17	M	44	24.7	Myalgia	43	172	Right VL	Unspecific changes
18	F	57	22.9	Acute muscle weakness	57	1563	Left VL	Necrotizing myopathy
19	M	30	32.8	Hyper‐CKemia, proximal muscle weakness	29	3984	Left GM	Necrotizing myopathy
20	M	57	24.5	Myalgia	56	1300	Left GM	Necrotizing myopathy
21	M	33	31.8	Hyper‐CKemia, myalgia	30	950	Right VL	Unspecific changes
22	M	41	35.2	Hyper‐CKemia, proximal muscle weakness	40	530	Left BF	Unspecific changes
23	M	48	26.9	Myalgia, stress intolerance	46	2592	Right TA	Neurogenic changes
24	M	36	24.7	Proximal muscle weakness	3	453	Left VL	Lipofibromatosis
25	M	59	30.9	Myalgia, muscle cramps	57	204	Left GM	Unspecific changes
26	M	24	26.0	Eye and proximal skeletal muscle weakness	13	1771	Left VL	Myopathic changes

Abbreviations: BF, biceps femoris; BMI, body mass index; CK, creatine kinase, GL, gastrocnemius lateralis; GM, gastrocnemius medialis; TA, tibialis anterior; VL, vastus lateralis.

### Muscle biopsy

The exact localization of the planned site of skeletal muscle biopsy was identified by pre‐biopsy MRI as well as during the operation procedure according to the macroscopic morphology of the tissue. In this process, a muscle with intermediate fatty and/or oedematous changes was chosen, to ensure obtaining tissue with typical histopathological changes. After application of general anaesthesia, a piece of about 1 × 1 cm^3^ was taken out of the designated muscle using an open biopsy technique. After the surgical procedure, skeletal muscle biopsies were divided into 0.5 cm^3^ samples oriented on a piece of cork under a magnifying glass to assure correct orientation of the muscle fibres and obtain cross‐sectioned fibres in later process. The tissue was embedded into tissue freezing medium (Leica Microsystems, Wetzlar, Germany) and snap frozen in liquid‐nitrogen‐cooled isopentane.

### Quantitative muscle MRI and processing

**FIGURE 2:**
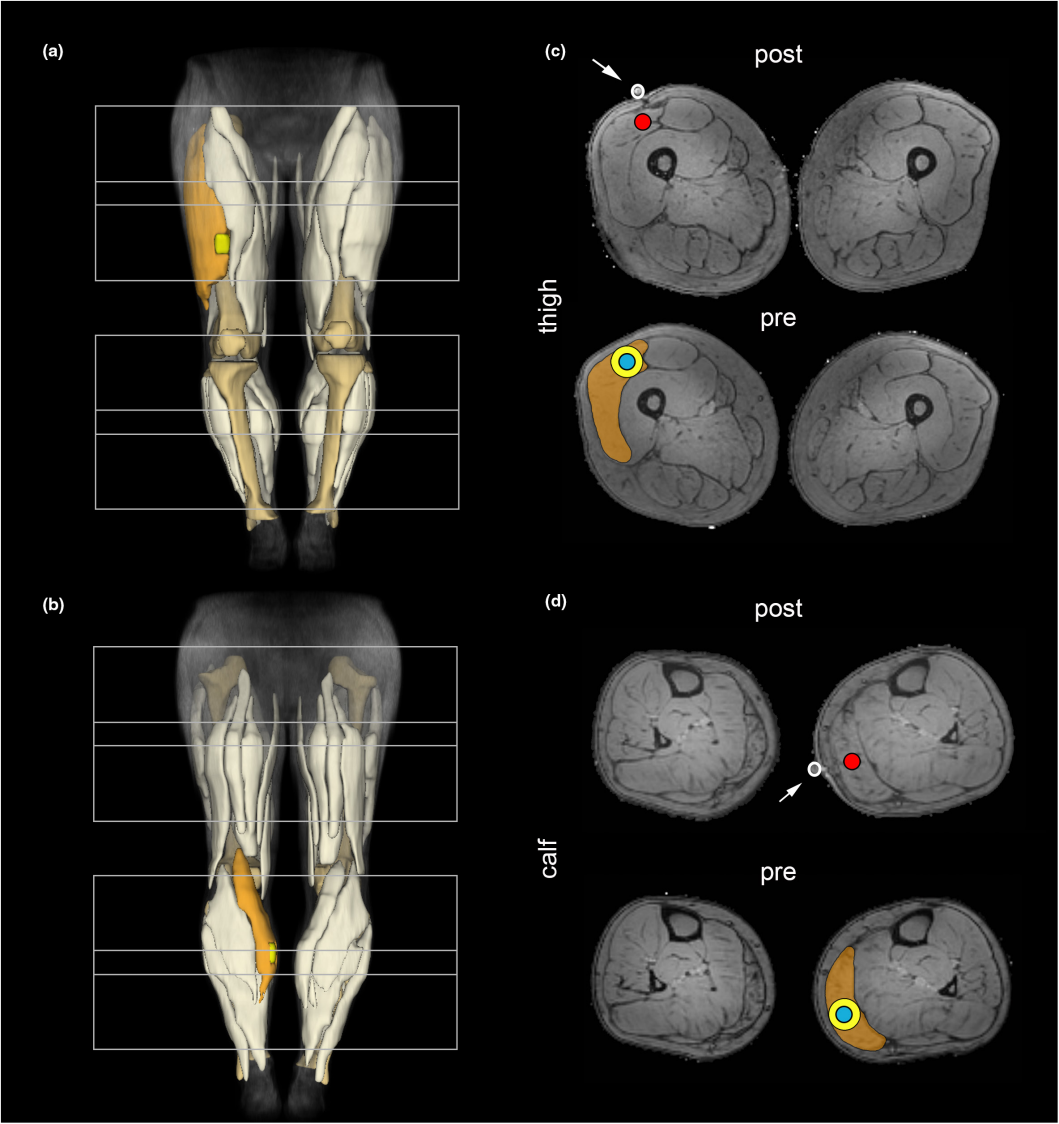
Overview of the field of view in thigh and calf ((a) front view; (b) back view). The different segmentation masks are highlighted in blue (6‐ mm radius around biopsy), yellow 10‐ mm radius around biopsy) and orange (whole muscle) for the classic muscle biopsy sites in representative participants ((a), (c) right vastus lateralis muscle; (b), (d) left gastrocnemius medialis muscle). Arrows denote the use of vitamin E pills for localization of the biopsy site (highlighted in red) in post‐intervention MRI.

Subjects were directed to maintain a stationary, supine position with their feet first, utilizing cushions to support their knees and sandbags placed around their feet to minimize any movement. A Philips 3.0 T Achieva MR system and a 16CH Torso XL coil were utilized to acquire scans from both legs, vertical to the femur and tibia bone. Figure 2 illustrates the fields of view (FOV), each measuring 480 × 276 ×150 mm^3^ in size (two FOV with an overlap of 3 cm for the thigh region). The imaging protocol included a four‐point Dixon‐based multi‐echo gradient‐echo sequence, a multi‐echo spin‐echo sequence and a diffusion‐weighted spin‐echo echo planar imaging (see Table S1) [23]. The total scanning time for the pre‐biopsy MRI was approximately 48 min. To identify the exact location of the biopsy, only the specific region containing the muscle biopsy was scanned in the second scan post‐biopsy. A vitamin E pill was used to identify the position of the FOV to perform the MR sequences in the correct location. The location and depth of the biopsy was afterwards visually determined on the high‐resolution MR images (see Figure 2).

The data underwent preprocessing as outlined in Schlaffke et al. using QMRITools (http://qmritools.com) [23, 24]. To summarize, denoising was applied to the diffusion data using a principal component analysis method. Subsequently, each leg underwent separate registration to correct for subject motion and eddy currents [25]. Dixon‐based data served as a target for b‐spline registration employed for echo planar imaging susceptibility correction. After tensor estimation, the diffusion metrics FA, mean diffusivity (MD), axial diffusivity (*λ*_1_) and RD were extracted voxel‐wise for each muscle. To evaluate the individual fat fractions from the Dixon‐based images, the IDEAL method was utilized to generate separate water and fat maps by considering a single T2* decay [26]. The derived water maps were used for the muscle segmentation. The T2‐mapping data were analysed using an extended phase graph fitting approach [27, 28].

The localization of muscle biopsy was segmented on pre‐biopsy images by an experienced rater (JF, 5 years of experience) using the post‐biopsy MRI as a reference. Whole muscles were segmented considering subcutaneous fat and fascia as termination voxels on all slices of the proton density image using the software package 3D slicer (3D‐slicer 4.4.0, https://www. slicer.org). For the analysis these segmentations were aligned to the other modalities (e.g., diffusion and T2 space) and subsequently eroded by one voxel. Three different segmentation masks were defined including the whole muscle volume, and two different sizes for the region of interest of the muscle biopsy. These regions of interest were established by creating a circular region with a 6‐ or a 10‐mm radius over five consecutive slices (see Figure 2). In the next step, the segmentation was registered to the diffusion space using sequential rigid and b‐spline transformations (elastix, http://elastix.isi.uu.nl) [29].

To extract the quantitative parameters within the three different masks, the masks were superimposed on the respective parameter maps (fat fraction, water T2, FA, MD, RD).

### Histological and immunofluorescence studies

Haematoxylin and eosin stainings were performed according to standard procedures [30]. Immunofluorescence studies were performed on muscle samples as described [31, 32]. Serial skeletal muscle cryosections of 10‐μm thickness were fixed, permeabilized, blocked and incubated overnight at 4°C with the primary antibodies listed in Table S2. All primary antibodies were diluted in 2% bovine serum albumin in phosphate‐buffered saline for immunofluorescence. Alexa Fluor 488‐conjugated goat anti‐mouse immunoglobulin (Ig) G (dilution 1:1000, #115–545‐146) and goat anti‐rabbit IgG (dilution 1:1000, #111–545‐144), goat anti‐mouse IgG2b human ads‐Alexa Fluor 488 (dilution 1:1000, #SBA‐1090‐30) and goat anti‐mouse IgG1 TRITC (dilution 1:1000, #ABIN 37652) were used as secondary antibodies.

Quantification of histological parameters was performed according to Güttsches et al. [20]. In brief, the area of fat tissue and the total cross‐sectional area of the muscle biopsy were measured in three fields of view in each muscle sample and the proportion of fat in the cross‐sectional area was calculated. Muscle fibre diameters of type 1 and type 2 muscle fibres were measured with an Olympus microscope (Olympus IX83, Germany). The minimal, maximal and mean diameters of each type 1 and type 2 muscle fibre were determined, as described before [20]. The degree of histopathological ‘vacuolar’ and ‘inflammatory’ alterations as well as the intensity of the immunofluorescence markers for major histocompatibility complex I (MHCI), CD3, CD68, p62, FYCO1 and myotilin were categorized into the three categories 0, 1 or 2 (0, normal; 1, slight to moderate increase; 2, strong increase).

### Statistical analysis

All statistical analyses were performed using IBM SPSS V28. To assess the influence of different segmentation masks repeated‐measures ANOVA was applied for each parameter. Furthermore, intra‐class correlation coefficients (ICCs) were calculated using a two‐way mixed design and a consistency definition to evaluate the reliability of qMRI metrics using different segmentation masks.

Subsequently, each qMRI parameter was correlated with its presumed corresponding histopathological parameter using Pearson correlation coefficients for metric values and Spearman correlation coefficients for ranked values, for example Dixon‐based FF with FF in cross‐section or water T2 relaxation time with inflammatory markers such as CD68‐positive macrophages. In one muscle biopsy specimen, correlation could only be performed regarding the FF in qMRI and the FF in skeletal muscle biopsy, as due to lipofibromatosis no further stainings could be performed. The significance level for all tests was set at *p* < 0.05. Variables were considered as ‘moderately correlated’ at correlation coefficients between *r* >0.3 and <0.5, as ‘well correlated’ between *r* >0.5 and <0.7 and as ‘strongly correlated’ between *r* >0.7 and 1 [33].

## RESULTS

### Muscle biopsies

Unspecific changes were found in eight participants of this study. Seven participants showed features of a myopathy or muscular dystrophy in histopathology (including two patients with a mitochondrial myopathy, one with a neuromyopathy, one with a degenerative vacuolar myopathy, one with a myofibrillar myopathy, one with lipofibromatosis and one with myopathic changes). Eight exhibited features of myositis, whilst two displayed the classic histopathological changes of sporadic inclusion body myositis. Neurogenic changes were found in one participant. An overview of the patients with clinical and histopathological features is given in Table 1. Five patient subgroups were defined: unspecific changes, degenerative myopathy, sporadic inclusion body myositis, inflammatory myopathy, and neurogenic changes.

### Influence of segmentation mask on qMRI metrics

An overview of the descriptive statistics of each qMRI parameter using the different segmentation masks is given in Table 2. No significant differences were found between the three approaches. ICC showed good to excellent reliability of qMRI metrics for different segmentation masks, ranging from 0.779 for MD to 0.997 for FF. Given the lack of significant differences and good reliability of qMRI metrics between segmentation masks, qMRI extracted from the small region of interest corresponding to the muscle biopsy site was used for correlation with histopathological findings.

**TABLE 2:** Overview of descriptive statistics of the qMRI metrics fat fraction (FF), T2 relaxation time, fractional anisotropy (FA), mean diffusivity (MD), axial diffusivity (*λ*_1_) and radial diffusivity (RD) for the different segmentations.

	biopsy (small; 6‐ mm radius)			biopsy (big; 10‐ mm radius)			whole muscle			*p* value	ICC
	Min.	Max.	Mean ± SE	Min.	Max.	Mean ± SE	Min.	Max.	Mean ± SE		
FF	3.21	78.29	8.84 ± 2.85	2.69	73.04	8.40 ± 2.65	3.59	73.52	8.64 ± 2.67	0.405	0.997
T2	27.13	38.65	31.06 ± 0.49	28.45	36.40	30.67 ± 0.32	26.01	38.09	30.93 ± 0.44	0.307	0.924
FA	0.128	0.384	0.239 ± 0.013	0.172	0.491	0.243 ± 0.015	0.142	0.340	0.229 ± 0.010	0.372	0.875
MD	1.133	1.721	1.504 ± 0.032	0.873	1.670	1.513 ± 0.034	1.339	1.759	1.541 ± 0.023	0.363	0.779
*λ*_1_	1.428	2.195	1.876 ± 0.040	1.265	2.093	1.903 ± 0.039	1.552	2.242	1.900 ± 0.034	0.560	0.821
RD	0.980	1.493	1.307 ± 0.033	0.828	1.456	1.333 ± 0.028	1.141	1.511	1.347 ± 0.024	0.169	0.894

*Note*: *p* values are derived from repeated‐measures ANOVA. ICCs were calculated using a two‐way mixed design and a consistency definition.

Abbreviation: ICC, intra‐class correlation coefficient.

The color shades were used to better distinguish the column groups representing the three segmentation masks used in the analysis. Alternatively, the blue shade could be displayed in grey and the orange shade in white. In this way, the groups of columns representing the segmentation masks can also be distiguished from each other at first sight.

### Correlation of FF with histopathological findings

**FIGURE 3:**
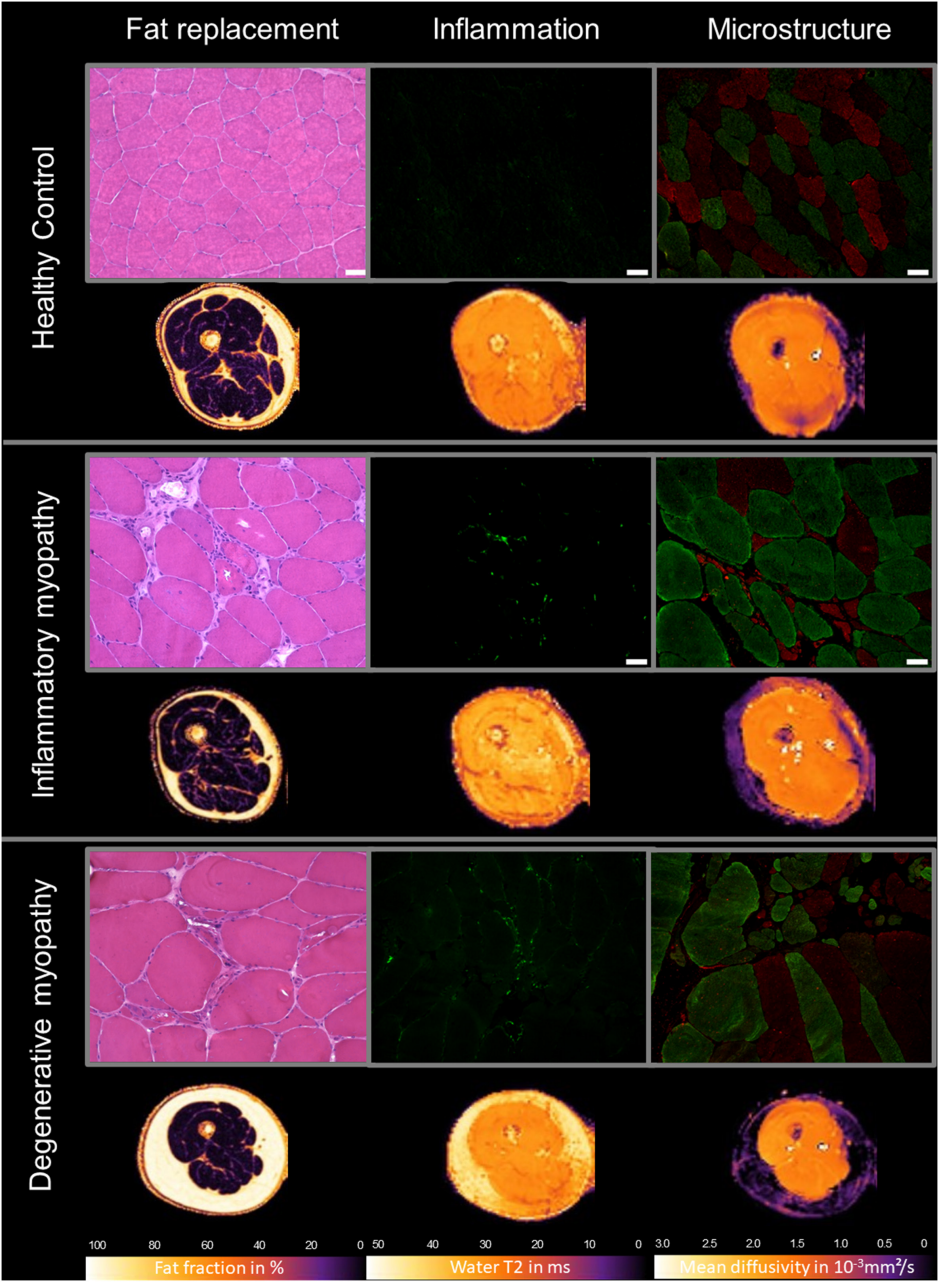
Skeletal muscle biopsy and corresponding qMRI parameter maps of representative participants with histopathological diagnosis of unspecific changes, inflammatory myopathy and degenerative myopathy.

**FIGURE 4:**
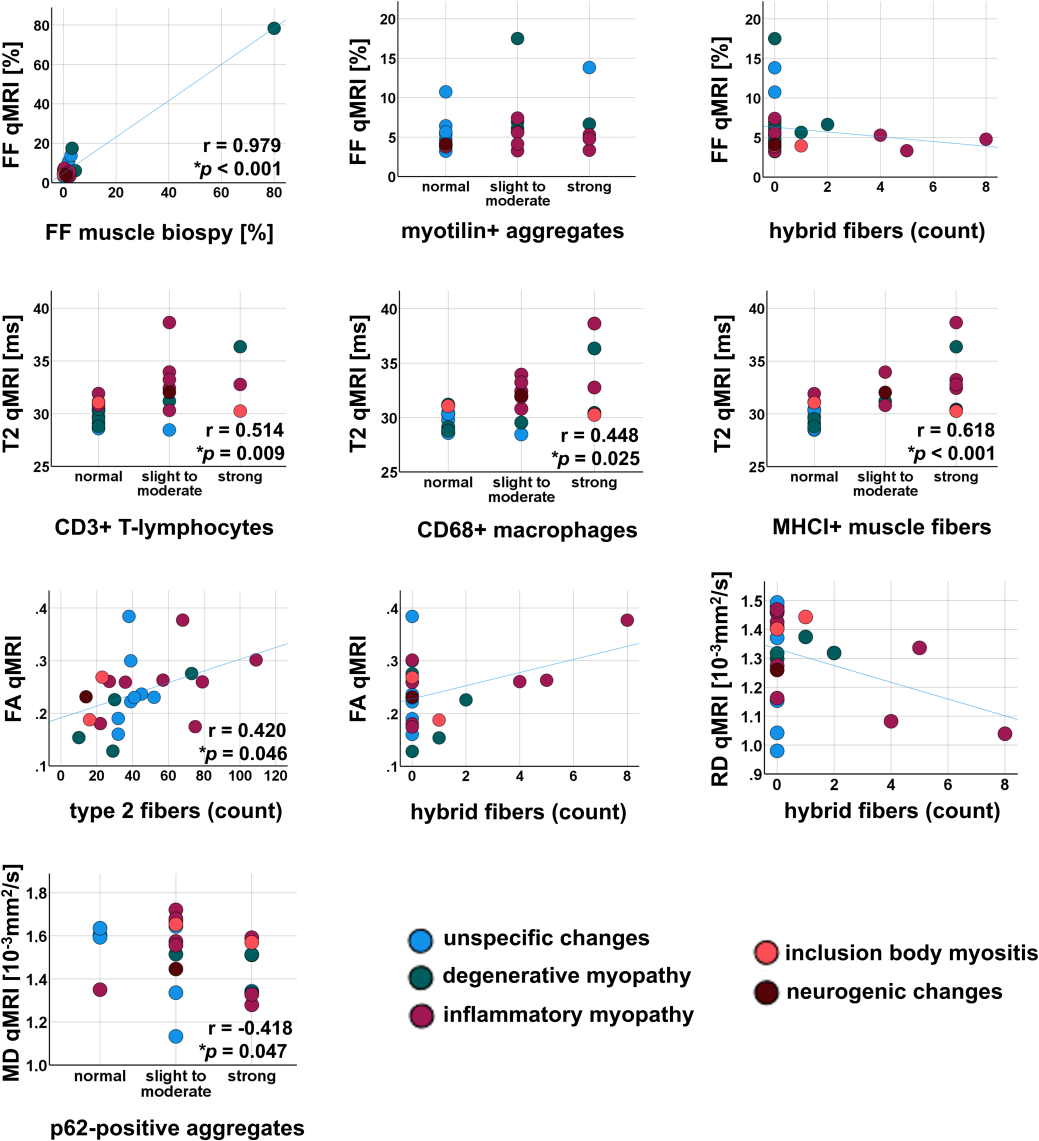
Correlations of qMRI parameters with histopathological findings in skeletal muscle biopsies. Correlation of FF to myotilin‐positive aggregates or fibre types is not available for the specimen with an FF of 80% due to severe fatty tissue degeneration. FF, fat fraction; T2, water T2 relaxation time; FA, fractional anisotropy; RD, radial diffusivity, MD, mean diffusivity, MHCI, major histocompatibility complex.

Figure 3 provides an overview of skeletal muscle biopsy findings and corresponding qMRI parameter maps. FF calculated from the qMRI data correlated strongly and significantly with the proportion of fat tissue in the skeletal muscle biopsy sample (*r* = 0.979, *p* < 0.001). Correlation was also significant, although less strong, when calculating it without including the highly fatty degraded muscle tissue (*r* = 0.447, *p* < 0.025). No significant correlations were found between FF and myotilin‐positive aggregates (*r* = 0.256, *p* = 0.217) and the number of hybrid muscle fibres (*r* = −0.177, *p* = 0.398) as a marker for the degeneration of muscle cells. No correlation of FF in qMRI to myotilin‐positive aggregates or fibre types could be calculated for the specimen with an FF of 80%, as stainings could not be performed in this highly degraded muscle tissue. Regarding the subgroups, patients with muscular dystrophy showed the highest FF in qMRI and on muscle biopsy (see Figure 4).

### Correlation of T2 with histopathological findings

Water T2 relaxation time, reflecting tissue oedema, was compared to typical histopathological features associated with inflammation. Good and significant positive correlations were found between water T2 and MHCI (*r* = 0.618, *p* < 0.001) and CD3‐positive T lymphocytes (*r* = 0.514, *p* = 0.009), as well as moderate but significant correlations between water T2 and CD68‐positive macrophages (*r* = 0.448, *p* = 0.025), suggesting that higher T2 is associated with inflammatory activity in histopathology (see Figure 4). Since structural defects of the muscle cells might result in tissue oedema, the numbers of vacuolar alterations of myofibres, p62 and FYCO1 as markers for tissue degeneration and autophagy were correlated with water T2. No significant correlations were found for water T2 with p62, FYCO1 or cytoplasmic vacuoles.

### Correlation of diffusion metrics with histopathological findings

The correlation analysis revealed a moderate but significant association between the quantity of type 2 fibres and FA (*r* = 0.420, *p* = 0.046). However, no significant correlations were observed with other diffusion metrics. Notably, the number of hybrid fibres demonstrated a trend towards a positive correlation with FA and a negative correlation with RD (FA, *r* = 0.397, *p* = 0.061; RD, *r* = −0.376, *p* = 0.077). Additionally, a moderately significant negative correlation of MD and p62 was observed (*r* = −0.418, *p* = 0.047).

## DISCUSSION

In this follow‐up study, qMRI parameters from the exact biopsy location were correlated with histopathological findings obtained by skeletal muscle biopsies. Former correlation analyses have been criticized because they correlated qMRI metrics of the whole muscle volume to histopathological findings in a specific region of the examined muscle [20]. Furthermore, some studies had a time latency between MRI and muscle biopsy of up to a month [19]. In comparison to those previous studies, in this study patients were investigated using qMRI shortly before and immediately (<24 h) after the skeletal muscle biopsy was taken [19, 20]. In that way, the exact localization of the biopsy could be pinpointed, and FF, water T2 relaxation time and the diffusion metrics of this localization to the histopathological results of the skeletal muscle biopsy sample could be correlated. Our data are in line with previous studies, showing that the qMRI parameters FF, water T2 and moderately FA, MD and RD correlate with histopathological findings [17, 18, 19, 20]. In this context, the size of the segmentation mask used for qMRI analysis had no influence on the correlation to the histopathological findings, indicating that all three approaches could be used for follow‐up.

The correlation of FF to the proportion of fat tissue in the skeletal muscle biopsy sample demonstrated across the cohort of patients in this study provides further evidence that quantification of muscular fat content by qMRI is suitable to reflect the process of lipomatous modification in neuromuscular disorders, in highly degraded muscle as well as in muscle tissue with beginning fatty degeneration. Dixon‐based sequence derived FF is thus an important tool in being more sensitive in the early detection of disease progression, in addition to clinical assessments [6, 34]. Due to the correlations with both histopathological and clinical assessments, FF is thus already used as a secondary endpoint in clinical trials [9, 35, 36].

Water T2 relaxation time is sensitive to changes in water content and mobility within tissue. Therefore, it has been proposed to be indicative of oedema, muscle fibre damage or inflammatory processes in general [11]. In this follow‐up study, it was possible to replicate the correlations of water T2 relaxation time with MHCI, CD3‐positive T lymphocytes and CD68‐positive macrophages, reflecting inflammatory histopathology [20]. These findings are in line with results in inflammatory myopathy, where water T2 relaxation time correlated with a previously defined score of inflammatory cells [19]. Absent correlations of water T2 relaxation time with vacuolar alterations of myofibres or markers for tissue degeneration and autophagy imply that changes in water T2 relaxation time seem to reflect inflammatory alterations primarily. Consequently, water T2 relaxation time could be considered a quantitative and non‐invasive marker of disease activity, especially in inflammatory conditions, supporting its potential in clinical contexts [37]. Its utility as a biomarker may aid therapy decisions by informing progression and guiding escalation or de‐escalation strategies.

Similar to water T2 relaxation time, diffusion metrics have been shown to reflect tissue damage, for instance post‐exercise [22]. Reduced FA and increased MD matched the histological indicators of damage using needle biopsies [22]. Recently, the fibre size measured in muscle biopsies has been correlated to the fibre size calculated based on diffusion metrics using the random permeable barrier model [14]. In patients with Becker muscular dystrophy larger fibre sizes were found in both diffusion‐weighted scans and histopathology. Still, they did not correlate in patients who underwent MRI and muscle biopsy, possibly due to a lack of colocalization of these methods [14]. These results cannot be transferred directly to the present study since Cameron et al. used comparably long diffusion times known to reflect fibre size better than the relatively short diffusion time used in this study [14, 38]. However, the positive correlation between the number of (hybrid) muscle fibres with the FA and the negative correlation with RD could indicate that diffusion metrics can reflect the existence of hybrid fibres. Hybrid muscle fibres, which are fibres with molecular features of both type 1 and type 2 fibres, are an unspecific finding and are more numerous when fibre shift occurs [39]. A fibre shift can be observed regularly in neuromuscular disorders, especially muscular dystrophies, or in muscle atrophy due to denervation [39, 40, 41]. Our findings propose that the described alterations of FA and RD reflect hybrid muscle fibre presence, possibly linked to a fibre shift as a hint at muscular degeneration [39]. Interestingly, an increase in FA in combination with a decrease in RD may signal early fibre atrophy in neuromuscular disorders which is also supported by simulations [16, 38, 42]. Recent work in mice suggested that diffusion changes measured with relatively short diffusion times could be explained by intracellular restrictions, for example due to an increased autophagic buildup [43]. Rohm et al. also showed significant negative correlations between MD and p62, an autophagic marker, which could be verified in humans in our study [43]. This could suggest that diffusion‐weighted scans may help in observation of changes in autophagic function. Overall, these preliminary results for histopathological mechanisms of diffusion metrics warrant further research to fully understand the underlying pathophysiological mechanisms of diffusion metrics.

In this study, patients were assessed shortly before and after the skeletal muscle biopsy to match qMRI and histology localization. The differences in qMRI parameters were assessed between three different sections of the muscle biopsied: the exact localization of the biopsied area (‘biopsy small’), a part including the area around the place of the biopsy (‘biopsy big’) and qMRI parameters of the whole muscle (‘whole muscle’; see Table 2). In our cohort, no significant differences between the different segmentation masks were found; thus it could be considered that qMRI measurements of the whole muscle could suffice to reflect the histopathological changes found in the muscle biopsy. However, this result may not be generalizable, as differences in FF along the proximo‐distal muscle axis has been reported [44, 45].

There are some possible limitations in this study. First, the qMRI techniques used are still non‐standard MR acquisition and need sophisticated postprocessing. Several different pipelines exist but harmonization of MR acquisition protocols and postprocessing between different centres is still challenging [11]. Second, the inclusion of different muscles, each with unique architectures, might have impacted fibre type and size analysis.

## CONCLUSION

In this study, significant correlations were shown of qMRI parameters for fat infiltration, inflammatory processes and fibre architecture properties with histopathological features in muscle biopsies. Importantly, the absence of significant differences between segmentation masks suggests that whole muscle qMRI could reliably represent histopathology, useful when post‐biopsy MRI is unavailable. Taken together, qMRI parameters could be validated especially for patient follow‐up, as a muscle biopsy is invasive and not suitable for this purpose. Given that qMRI parameters are further refined, they could also be considered to partly replace skeletal muscle biopsy; however, further studies are needed in this regard.

## AUTHOR CONTRIBUTIONS

**Anne‐Katrin Güttsches:** Conceptualization; investigation; writing – original draft; writing – review and editing. **Johannes Forsting:** Investigation; data curation; formal analysis; writing – original draft; writing – review and editing. **Moritz Kneifel:** Investigation. **Robert Rehmann:** Investigation. **Alice De Lorenzo:** Investigation. **Elena Enax‐Krumova:** Investigation; writing – original draft; writing – review and editing. **Martijn Froeling:** Data curation; formal analysis. **Matthias Vorgerd:** Investigation; writing – review and editing. **Lara Schlaffke:** Conceptualization; investigation; data curation; formal analysis; writing – original draft; writing – review and editing.

## CONFLICT OF INTEREST STATEMENT

The authors of this manuscript declare no relationships with any companies, whose products or services may be related to the subject matter of the article.

## Supporting information


**Table S1.** Scan parameters for the acquired Dixon sequences, quantitative T2 and diffusion‐weighted imaging (DWI).

**Table S2.** Primary and secondary antibodies used in immunofluorescence studies.

## Data Availability

The data that support the findings of this study are available from the corresponding author upon reasonable request.
