# Supplementary material for: Pre‐ and post‐skeletal muscle biopsy quantitative magnetic resonance imaging reveals correlations with histopathological findings
Source: Eur J Neurol. 2024 Sep 16;31(12):e16479. doi: 10.1111/ene.16479 (PMC11555129; doi:10.1111/ene.16479)
Supplement: Supplementary file 1 — Table S1. Scan parameters for the acquired Dixon sequences, quantitative T2 and diffusion‐weighted imaging (DWI). [file ENE-31-e16479-s001.docx]

**Supplementary Table S1:** Scan parameters for the acquired Dixon sequences, quantitative T2, and diffusion weighted imaging (DWI).

|  | qT2 | DWI | Dixon |
| --- | --- | --- | --- |
| Sequence | MESE | SE-EPI | MS_FFE |
| FOV (Lr x AP x FH mm^3^) | 480 x 276 x 150 | | |
| Acquisition matrix | 160 x 92 | 160 x 92 | 320 x 184 |
| Voxel size (mm^3^) | 3 x 3 x 6 | 3 x 3 x 6 | 1.5 x 1.5 x 6 |
| Slice gap (mm) | 6 | - | - |
| Slices | 13 | 25 | 25 |
| TR (ms) | 4598 (7650) | 5000 | 210 |
| TE (ms) | 17 x Δ 7.6 | 57 | 2.6/3.36/4.12/4.88 |
| Flip angle (°) | 90/180 |  | 8 |
| Sense factor | 2 | 1.9 | 2 |
| b‐values (number of images) | 0 | 0 (1), 1 (6), 10 (3), 25 (3), 100 (3), 200 (6), 400 (8) and 600 (12) | 0 |
| Fat suppression | - | SPAIR/SPIR | - |
| Total duration (min) | 3.08 | 4.33 | 1.33 |
