# Supplementary material for: Pre‐ and post‐skeletal muscle biopsy quantitative magnetic resonance imaging reveals correlations with histopathological findings
Source: Eur J Neurol. 2024 Sep 16;31(12):e16479. doi: 10.1111/ene.16479 (PMC11555129; doi:10.1111/ene.16479)
Supplement: Supplementary file 2 — Table S2. Primary and secondary antibodies used in immunofluorescence studies. [file ENE-31-e16479-s002.docx]

**Supplementary Table S2:** Primary and secondary antibodies used in immunofluorescence studies.

| **Antigen** | **Source** | **Company** | **Number** | **Dilution** |
| --- | --- | --- | --- | --- |
| Major histocompatibility complex I (MHC I) | mouse (mAb) | Dako | #M0736 | 1:100 |
| CD3 | mouse (mAb) | Dako | #M7254 | 1:50 |
| CD68 | mouse (mAb) | Dako | #M0718 | 1:200 |
| p62 | mouse (mAb) | Abcam | #ab56416 | 1:1000 |
| FYCO 1 | rabbit (pAb) | Sigma | #HPA035526 | 1:500 |
| myotilin | mouse (mAb) | Novocastra | #NCL-MYOTILIN | 1:20 |
| myosin heavy chain slow (type 1-muscle fibres) | mouse (mAb) | Hybridoma Bank | #BA-F8 | 1:30 |
| myosin heavy chain 2A (type 2-muscle fibres) | mouse (mAb) | Hybridoma Bank | #SC-71 | 1:20 |

*mAb - monoclonal antibody, pAb - polyclonal antibody.*
